# Metabolite-derived protein modifications modulating oncogenic signaling

**DOI:** 10.3389/fonc.2022.988626

**Published:** 2022-09-23

**Authors:** Yawen Liu, Anke Vandekeere, Min Xu, Sarah-Maria Fendt, Patricia Altea-Manzano

**Affiliations:** ^1^ Department of Gastroenterology, Affiliated Hospital of Jiangsu University, Jiangsu University, Zhenjiang, China; ^2^ Laboratory of Cellular Metabolism and Metaboli Regulation, VIB-KU Leuven Center for Cancer Biology, VIB, Leuven, Belgium; ^3^ Laboratory of Cellular Metabolism and Metabolic Regulation, Department of Oncology, KU Leuven and Leuven Cancer Institute (LKI), Leuven, Belgium

**Keywords:** tumor suppressor gene, post-translational modification, metabolites, tumor microenvironment, oncogenic signaling

## Abstract

Malignant growth is defined by multiple aberrant cellular features, including metabolic rewiring, inactivation of tumor suppressors and the activation of oncogenes. Even though these features have been described as separate hallmarks, many studies have shown an extensive mutual regulatory relationship amongst them. On one hand, the change in expression or activity of tumor suppressors and oncogenes has extensive direct and indirect effects on cellular metabolism, activating metabolic pathways required for malignant growth. On the other hand, the tumor microenvironment and tumor intrinsic metabolic alterations result in changes in intracellular metabolite levels, which directly modulate the protein modification of oncogenes and tumor suppressors at both epigenetic and post-translational levels. In this mini-review, we summarize the crosstalk between tumor suppressors/oncogenes and metabolism-induced protein modifications at both levels and explore the impact of metabolic (micro)environments in shaping these.

## Introduction

Cancer initiation and progression are characterized by a series of genetic and epigenetic alterations (1). As such, mutations inducing a gain-of-function in oncogenes or a loss-of-function in tumor suppressor genes (TSGs) are able to adjust the cell cycle boundaries and transform a mammalian cell into its malignant derivative (2, 3). As a result, the cells maintain continuous growth thanks to aberrant oncogenic signaling and the inactivation of cell cycle suppressors (4, 5).

To fulfill the energy and bio-mass demands of continuous proliferation, malignant cells acquire the capacity to rewire their metabolism (6). During this reprogramming, tumors rely on specific metabolic pathways depending on extrinsic factors like the site of tumor growth, as well as intracellular signals (7). Previously, mutations in oncogenes and TSGs have been linked to an altered metabolic phenotype, with the Warburg effect – the preference for glycolysis even under sufficient oxygen supply – as an example of metabolic reprogramming regulated by oncogenic signaling (8–12). Nevertheless, while oncogenic signaling reprograms cellular metabolism, also metabolites and metabolic enzymes themselves are able to modulate the activity of oncogenic proteins in a bi-directional feedback loop (13).

One way by which the function of an (onco)protein can be affected by metabolites is through protein modifications, a process in which a small chemical substrate (such as lactyl-CoA, palmitoyl-CoA, acetyl-CoA, succinyl-CoA) is covalently bound to a protein of interest (14). Depending on the type and dynamics of the modification, this changes the transcription of oncogenes or their protein stability, interaction, localization and overall activity (14). Many of the attached substrates are derived from central carbon metabolites, hereby linking the metabolic phenotype to downstream protein modifications (13). Consequently, changes in the metabolism and its derived metabolites can alter the modifications during cancer progression and thus the downstream regulation of the protein signaling cascade.

A tumor does not only consist of transformed cancer cells, but contains a complex entity of heterogeneous cell types which has been referred to as the “tumor microenvironment” (TME) (15). This TME displays a differing nutritional and metabolite composition depending on the intracellular metabolism of the various cell types, the cell-cell interactions and the specific tumor location in the body (16, 17). This change in nutrient availability will not only directly affect the fuels used for cellular proliferation, but will also alter metabolite-derived protein modifications. Therefore, the type and extent of the modifications will depend on the environment in which the cancer cells site (18).

In this mini-review, we highlight a select number of established and emerging metabolite-derived protein modifications by which epigenetic changes related to oncogenes/TSGs or modulation of oncogenic protein products can be induced; and explore the role of the metabolic microenvironment in shaping these modifications.

## Lactylation

While a tumor grows, the inner center becomes deprived of oxygen. This phenomenon, called hypoxia, induces direct metabolic, transcriptomic and genetic changes within multiple cell types in the TME (8, 19). Both the lack of oxygen and the Warburg effect push the cancer cells to mainly rely on glycolysis, produce high levels of lactate and even excrete the metabolite into the extracellular space. This lactate has long been considered a metabolic waste product, but gained more notice after the discovery of several novel physiological functions in past years. Indeed, lactate is now known as a fuel for mitochondrial metabolism and as immune regulator through impairing cytokine production and polarization (20–25). However, the study of lactate function at molecular level is still a largely unexplored area.

**Figure 1:**
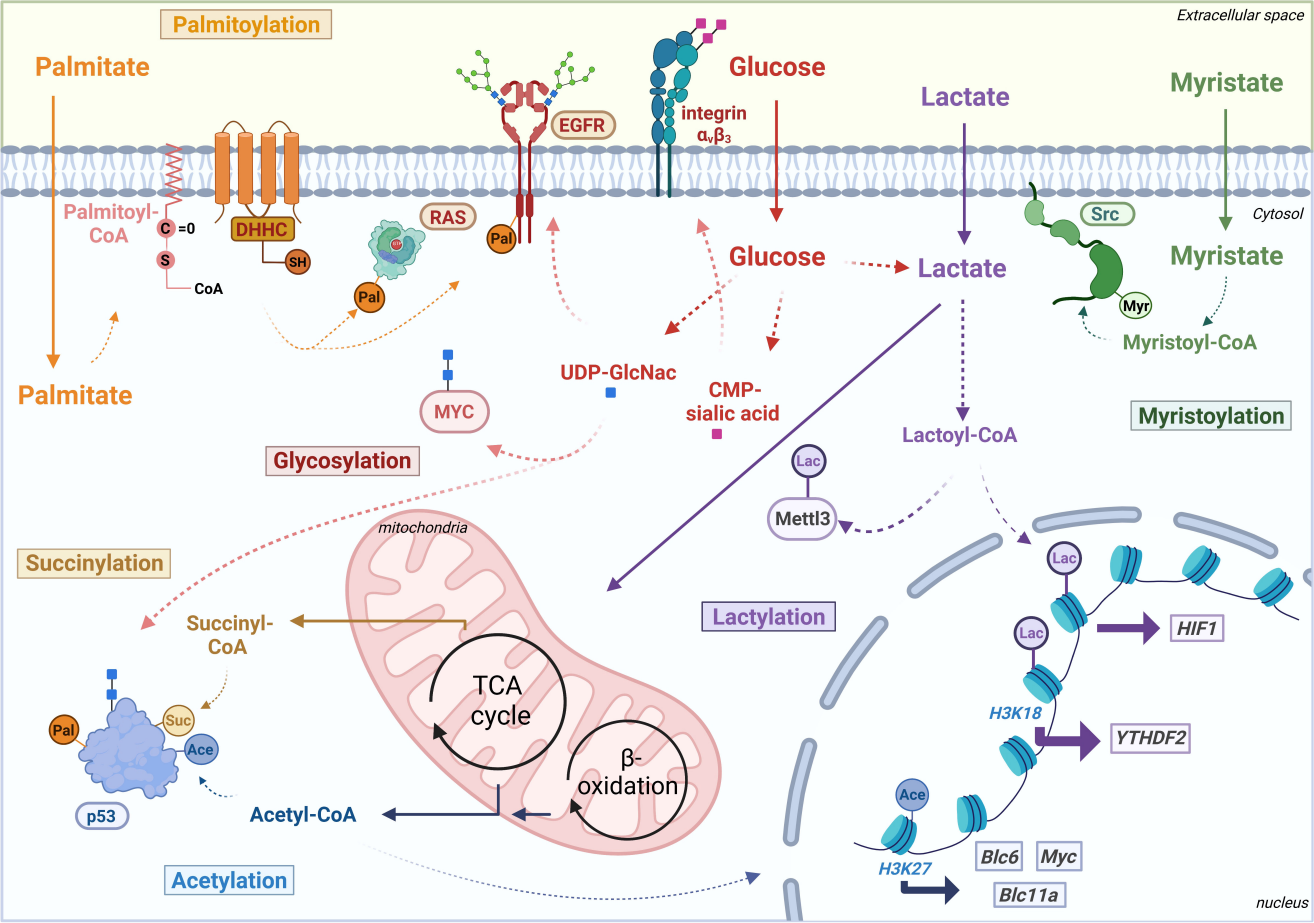
Schematic overview of metabolite-derived modifications palmitoylation (Pal), succinylation (Suc), glycosylation: O-GlcNAcylation (blue squares) and sialylation (pink squares), acetylation (Ace), lactylation (Lac) and myristoylation (Myr) affecting various oncogenes and TSGs at epigenetic level or post-translational level. Transcriptionally regulated oncogenes are put in squares and oncoproteins or tumor suppressor proteins in ovals. Thick arrows indicate transcriptional induction. B-cell lymphoma 6 protein (Bcl6), B-cell lymphoma 11a protein (Bcl11a), cytidine monophosphate sialic acid (CMP-sialic acid), Coenzyme A (CoA), Epidermal growth factor receptor (EGFR), hypoxia-inducible factor 1 (HIF1), Methyltransferase 3 (Mettl3), tricarboxylic acid (TCA), uridine diphosphate N-acetylglucosamine (UDP-GlcNAc), YTH domain-containing family protein 2 (YTHDF2). Figure created with Biorender.

Next to its conventional role in central carbon metabolism, lactate and its derived acyl-coenzyme A (CoA) - lactyl-CoA - have been shown to modify histone proteins in a process called lactylation (26). In this novel protein modification, lactic acid from exogenous and glucose-dependent endogenous sources is covalently bound to a lysine residue in histone proteins and directly affects gene transcription in multiple cancer cell types and macrophages under hypoxic conditions (26). Over the past few years, a growing number of studies show a strong association between histone lactylation and oncogenic signaling to induce the progression of human cancers (27, 28). Accordingly, elevated levels of global and specific H3K18 histone lactylation in ocular melanoma tissues have been associated with poor patient prognosis, which could be related to the transcriptional levels of the novel oncogene YTH N6 methyladenosine RNA binding protein *(YTHDF2)* in tumor-initiating myeloid cells (**Figure 1**) (29). Lactylated *YTHDF2* recognizes efficiently the m6A modification sites in the mRNAs of TSGs such as *PER1* and *P53* and then induces their degradation to accelerate tumor progression of ocular melanoma. Genetically or pharmacologically impairing this histone lactylation of *YTHDF2* not only inhibit proliferation and migration *in vitro*, but also reduces tumor size of orthotopic xenografts of ocular melanoma, reflecting the relevance of this modification *in vivo* (29).

Not only deprivation of oxygen, also the general availability of exogenous lactate can induce histone lactylation in a dose-dependent manner, highlighting the role of the metabolic microenvironment in inducing this protein modification (26, 27, 30). Indeed, in tumor-infiltrating myeloid cells – a key population involved in tumor immune escape – environmental lactate was shown to increase the transcriptional expression of oncogene methyltransferase like 3 *(METTL3)* through lactylation of histone H3K18. Remarkably, they found that lactate also directly targets two lysine residues in the METTL3 protein, which is highly essential for its activity to target RNAs and add m^6^A-modifications (**Figure 1**). By inserting these modifications in the mRNA of *JAK1*, lactylated *METTL3* strengthens the pro-tumoral JAK/STAT3 pathway and enhances tumor progression of colon cancer both *in vitro* and *in vivo* (31).

In non-small cell lung carcinoma (NSCLC), which has been shown to highly take up lactate for energetic purposes *in vitro* and in patients, this metabolite can act in two ways to aid cell survival (28). First of all, lactate fuels the tricarboxylic acid (TCA) cycle in the mitochondria to generate energy in terms of ATP. Next to that, lactate can activate the downstream transcription of hypoxia inducible factor 1 *(HIF-1)* through lactylating the promoter regions of glycolytic enzymes like hexokinase 1 (HK-1). As HIF-1 signaling is known to support the glycolytic phenotype and induce lactate generation, lactate thus stimulates its own regeneration by acting as transcriptional regulator (28, 32). All of this illustrates the link between a nutritionally enriched environment and epigenetic regulation of oncogenic signaling through nutrient-derived protein modification. However, more research will be needed to gain a comprehensive understanding of how environmental lactate stimulates cancer growth through (histone) lactylation of oncogenes.

## Protein lipidation

Upon measuring interstitial fluid of mice and patient-derived melanoma xenografts, marked increases in various free fatty acid (FA) species were seen, suggesting dynamic changes in lipid composition in the TME (33). Indeed, numerous studies have indicated the uptake of exogenous FAs and lipids by cancer cells to boost their tumor growth *in vitro* and *in vivo* (33–37). It is widely accepted that lipids and FA metabolism provide an important source of energy in cancer cells. However, other roles for fatty acids and their by-products are emerging in literature (38). In that regard, fatty acids palmitate and myristate have been shown to associate with proteins in order to enhance their hydrophobicity, protein-protein interactions and improve protein folding and stability (39). This attachment of lipid species is termed protein lipidation and comprises an essential class amongst the metabolic post-translational modifications (PTMs) (40).

## S-Palmitoylation

Attachment of palmitoyl-CoA - the acyl-CoA derived from palmitate - occurs through a thioester bond with a cysteine residue in the protein of interest (39, 40). This process called S-palmitoylation is a key feature for protein functionality and its imbalance can induce detrimental consequences in terms of cellular malignancy (41, 42). Transfer of the palmitoyl-CoA occurs enzymatically through a family of zinc finger DHHC-containing palmitoyl S-acyltransferases (DHHCs), of which multiple have been implicated in various cancer types like renal, pancreatic, ovarian and gastric cancer (40, 43, 44).

As S-palmitoylation drives protein stability, this lipid-derived protein modification can affect oncogenic and tumor suppressor protein products to promote cancer growth. For example, signaling of tyrosine kinase EGFR – one of the essential drivers of oncogenic signaling in various types of lung cancer – is amplified and prolonged thanks to its palmitoylation by transferase DHHC20 (**Figure 1**) (45). Since EGFR is known to mitigate its signaling through PI3K-Akt and RAS, loss of DHHC20 or a palmitoyl-resistant EGFR significantly reduces their oncogenic signaling and downstream *Myc* in a mouse model of oncogenic KRAS-driven lung adenocarcinoma and subsequently affects tumor growth both *in vitro* and *in vivo* (45, 46).

Other well-known example of oncogenes regulated by S-palmitoylation is the GTPase RAS (**Figure 1**), which is more explicitly discussed in the mini-review from Busquets-Hernandez et al (47). With RAS proteins being amongst the most frequently altered oncogenes in various human cancers, targeting their palmitoylation might be an effective therapy for multiple cancer types. As such, mice transplanted with bone marrow cells expressing the palmitoylation-deficient NRAS mutant remained without malignant growth for two years, while the wild-type oncogene limited survival to a maximum of 3 months upon developing a fatal myeloid leukemia-like disease (48). Also, recently, it was shown that members of the α/β hydrolase domain 17 family (ABHD17) family efficiently depalmitoylate NRAS (removal of the palmitoyl-CoA group). Reducing the expression of the ABHD17 family or treatment with a potent new pan-inhibitor – ABD957 – which targets all family members both impairs N-RAS signaling and hereby tumor growth in AML and immortalized kidney cells (49, 50). Therefore, aiming at the palmitoylation/depalmitoylation cycle of oncogenes provide a novel opportunity to target cancer cells that are dependent on NRAS for their growth (50).

Tumor suppressors can also be affected by S-palmitoylation. It was shown that S-palmitoylation of tumor suppressor p53 promotes its nuclear translocation and subsequent signaling (51). However, multiple *in vitro* cancer cells carrying a wild-type p53 are able to circumvent this PTM by recruiting epigenetic regulators to the promoter region of palmitoyltransferase DHHC1 (51). By doing this, cancer cells can overcome the tumor suppressive activity of p53 and promote their own progression (51). In addition, palmitoylation of protein GNA13 – a TSG commonly mutated in germinal center B-cell-like diffuse large B-cell lymphoma (GCB-DLBCL) and Burkitt’s lymphoma – was shown to be essential for its protein stability, membrane association and tumor suppressor activity (52). Interestingly, it was recently shown that GNA13 negatively correlates with the expression of cell death regulator BCL2 in a palmitoylation-dependent manner in these GCB-DLBL cells. By affecting the palmitoylation of GNA13, these cancer cells with a wild-type GNA13 protein became again sensitized to a BCL2 inhibitor, suggesting GNA13 palmitoylation as potential target for combinatory therapy with BCL2 inhibitors (53).

Another example of a tumor suppressor protein whose palmitoylation is essential for its activity is Scribble (SCRIB), a protein critical in cell polarity and cell-cell junctions (40, 54). S-palmitoylation by DHHC7 is essential for its efficient plasma-membrane targeting and subsequent tumor suppressive activities. Indeed, upon loss of its palmitoyl group, downstream oncogenic pathways of the Hippo pathway such as Yes-associated protein (YAP), MAPK and PI3K/Akt take over and promote tumorigenesis in immortalized kidney, ovarian and breast cancer cell lines (40, 54).

With palmitate being one of the most abundant FAs, an increased abundance and uptake within the TME could have a direct impact on the S-palmitoylation of target proteins in the various cell types. Even though this environmental link has not been described yet in cancer models, an increase in palmitate abundance due to diet or disease induces hyper-palmitoylation in hippocampal neurons and hepatocytes of mice (55–57). Therefore, considering the key role of palmitoylation in regulating protein localization and function and a marked increase of FAs in TME, further work investigating the relationship between these two features will arise.

## Myristoylation

Like palmitate, myristate and its derived myristoyl groups (14-carbon saturated fatty acyl groups) can be covalently attached to N-terminal glycine residues of proteins (58, 59). This lipidation called myristoylation is also critical for protein localization and stability, and it has been linked to various malignancies such as ovarian, lung cancer and leukemia (60, 61).

Lipidomics analysis of metastasizing ovarian cancer cells identified specifically myristic acid as being highly enriched compared to non-metastatic cells (60). Indeed, the increased myristic acid abundance was seen to enhance ovarian cancer in both mice and patients’ samples by inducing myristoylation of the oncogenic SRC pathway (**Figure 1**) (62, 63). Src and Src family kinases (SFKs) are proto-oncogenes that play a key role in regulating various cell surface signaling, and SFK myristoylation was shown to induce attachment to the cytoplasmic membrane and improvement their kinase activity (62, 63). Next to ovarian cancer, also prostate cancer progression can be affected through inhibition of SRC myristoylation, highlighting its importance in various cancer types (64). Myristoylation plays an important role in the *in vivo* growth of lung cancer, by affecting both tumor suppressor FUS1 and oncogene methyl transferase EZH2 (65, 66). While myristoylation of FUS1 is highly essential for its ability to suppress *in vivo* tumor growth, myristoylated EZH2 was shown to efficiently bind with STAT3 and promote *in vivo* lung cancer (67).

## Acetylation

Multiple nutritional sources (glucose, fatty acids, acetate, amino acids, etc) can generate acetyl-CoA, a metabolic intermediate carbon source with a key role in mitochondrial energetic purposes and lipid biosynthesis (68). Next to its metabolic role, acetyl-CoA can act as a substrate for acetylation, a reversible protein modification in which an acetyl group from acetyl-CoA is linked to lysine residues in histone or non-histone proteins. This modification, catalyzed by lysine acetyltransferases (KATs, formerly termed histone acetyltransferases or HATs), has been shown to contribute to cancer development and progression through the regulation of gene transcription (69–71). Indeed, cancer cells maintain their oncogenic signaling through acetylation of their epigenome, with the aberrant expression of *MYC*, *BCL6* and *BCL11A* induced by acetylation of histone H3 lysine 27 (H3K27) in lymphoma as an example (**Figure 1**) (72).

While histone acetylation has been proven to regulate the expression of some oncogenes (72–74), recent studies have shown that KATs are also capable of acetylating non-histone proteins at post-translational level, including a large number of oncogenes or TSGs (75, 76). It has been long known that acetylation is an essential regulator of p53 protein to stabilize and support its DNA-binding activities (77, 78). While its importance is extensively described in other reviews (77, 79, 80), Cao et al. recently observed an unexpected role for acetylated p53 in promoting *PD-1* (programmed cell death protein 1) expression in tumor cell lines of different origins, including lung cancer, osteosarcoma, melanoma, and pancreatic cancer (81). Once acetylated, p53 facilitates *PD-1* transcription by recruiting acetyltransferases onto its promoter. This regulation seems to be dependent on the two specific acetylation sites (K120/164) in the p53 protein, suggesting that acetylation at specific sites can determine the activity of a protein.

Regarding oncogenes, studies showed evidence of both direct and indirect acetylation of c-Myc signaling. For example, the oncoprotein MYC in T-cell leukemia is directly acetylated, while acetylation through histone H3K9 also indirectly elevated MYC transcription in neuroblastoma cells and hepatocarcinoma (82, 83). Indeed, many interacting cofactors of MYC possess acetyltransferase activity and modify different lysine residues in the oncoprotein during their interaction (84–86). As lysine can be a binding dock for both ubiquitinylation and acetylation, these modifications can interfere with one another. Therefore, direct acetylation prevents MYC from ubiquitin-mediated degradation and stabilizes the protein (85–87).

Remarkably, protein acetylation is highly sensitive to changes in acetyl-CoA levels and local changes in its intracellular compartmentalized abundance can impact acetylation at the specific intracellular compartments of the cell (cytosol versus nucleus) (88, 89). In this regard, even though acetyl-CoA itself cannot be taken up from the environment, differing availabilities from its sources can directly impact the intracellular acetyl-CoA abundance and its derived modification *in vivo* (90–92). Accordingly, acetate can not only act as bioenergetic substrate but can also function as an epigenetic regulator by enhancing histone acetylation of *Twist* - an inducer of EMT - in hepatocellular cell lines and patient samples, suggesting a plausible link between environmental acetate and intracellular acetylation (93–95). However, further studies are needed to evaluate the direct regulation of oncogenic signaling by environmentally induced acetylation. It is interesting to mention that immortalized astrocytes with tumor suppressor deficiency have marked upregulated expression of nucleo-cytosolic acetyl CoA synthetase *(ACSS)* enzymes that oxidize acetate to acetyl-CoA, suggesting the usage of acetate as carbon source in oncogenic settings (94). However, if they use acetate-derived acetyl-CoA for its protein modification remains elusive.

## Succinylation

Gastrointestinal stromal tumors, renal, thyroid, testicular tumors and neuroblastomas are often characterized by germline mutations in succinate dehydrogenase (SDH) enzyme. As a key metabolic enzyme in the TCA cycle, its deficiency results in an accumulation of succinate both intra- and extracellularly (96). As succinate can be immediately interconverted to succinyl-CoA, its increase will significantly affect succinyl-CoA levels as well as its derived succinylation (97). Succinylation is a PTM, in which succinyl groups are being dynamically and reversibly attached to lysine residues of proteins (98). The major substrate for succinylation is succinyl-CoA – a cofactor that can be generated from the mitochondrial TCA cycle, lipid and amino acid metabolism. This PTM has been shown to play an important regulatory role in the progression of a variety of tumors like thyroid, gastric and breast cancer (98, 99). In short, succinylation was seen to boost proliferation of both thyroid and breast cancer cells and promote metastasis in gastric cancer. Remarkably, while this PTM can promote tumorigenesis in those types of cancer, it has a tumor suppressive effect on liver cancer, lung cancer and osteosarcoma (100–102).

Mass spectrometry identified more than 500 succinylation sites in about 300 proteins in gastric cancer tissues, highlighting its importance in regulating cancer progression (99, 103). Next to the above-mentioned modifications of TSG p53, succinylation was added very recently to the list of its regulatory PTMs. Using mass spectrometric analysis, p53 was seen to be succinylated at lysine 120 (K120) which is also a common acetylation site and key modulatory residue (**Figure 1**). Loss of this modification by SIRT5 highly affects the p53 response to DNA damage and all gene expressions related to apoptosis and cell cycle arrest (p21, MDM2, TIGAR, SFN), indicating the importance of this PTM for its functionality (104). Nevertheless, whether this is related to any specific type of cancer remains elusive.

## Glycosylation

Glycosylation the addition of sugar chains to proteins or lipids - is one of the main features of malignancy and shows a glycosylation-specific gene expression during cancer progression (105–108). The enzyme O-linked N-acetylglucosamine transferase (OGT) involved in attaching the substrate to the protein and modification of the substrate O-linked β-N-acetylglucosamine (O-GlcNAc) itself are known to be necessary for tumorigenesis and metastasis abilities of multiple cancer types, like papillary thyroid cancer *in vivo*, breast, colon, liver and lung cancer (109–112).

Similar to the other PTMs described above, oncogenes and tumor suppressors can also be glycosylated to alter their activity (113, 114): EGFR family, estrogen receptor (ER) family, c-MYC, YAP, B-catenin, TSG protein retinoblastoma (Rb), p53 and others (**Figure 1**) (114–120). Indeed, O-glycosylation at threonine 58 in oncogene c-Myc was shown to be a major mutation site in human lymphomas and was shown to stabilize the protein to promote proliferation and tumorigenesis of hepatocellular carcinoma cells *in vivo* and *in vitro* (121–123). Moreover, oncoprotein YAP is stabilized by O-GlcNAcylation in both *in vitro* and *in vivo* models to promote high glucose-induced liver tumorigenesis (120). Similar, to other PTMs, oncogenes themselves can induce O-GlcNAcylation and OGT expression directly as part of their oncogenic signaling, indicating the existence of a bi-directional feedback loop between glycosylation and oncogenic expression (124).

Often, glycans and glycosylated proteins are located at the extracellular side of the plasma membrane. Also, any changes in the intracellular and microenvironmental metabolite pools can have a direct effect on O-GlcNAcylation, of which its substrate UDP-GlcNAc is produced through the hexosamine biosynthetic pathway. As glucose, amino acid, fatty acid and nucleotide metabolism are all linked to this pathway and therefore the synthesis of the substrate, any difference in metabolite levels from these pathways might influence O-GlcNAcylation (110). This highlights the possibility of a direct role for the microenvironment in the interplay between glycosylation and various cell types of the TME. Indeed, the glycolytic phenotype induced by loss of p53 was shown to elevate O-GlcNAc levels and its derived PTM in mouse embryonic fibroblasts and human transformed fibroblasts *in vitro* (125). A more detailed description of these dynamics can be found in the review Peixoto et al. (2019) (126).

Interestingly, very recently it was shown that glycosylation can be modulated by the heterogeneous protein expression of the metabolic enzyme phosphoglycerate dehydrogenase (PHGDH), which affects breast cancer-derived metastasis formation (111). Mechanistically, loss of PHGDH was shown to induce the activation of the hexosamine-sialic acid pathway and hereby the sialylation – a specific type of glycosylation - of integrin α_v_β_3_ (**Figure 1**). Doing this, the PHGDH_low_ breast cancer cells promote the migration and metastatic dissemination from the primary tumor (111). However, more research will be needed to not only fully elucidate the environmental role in inducing O-GlcNAcylation of oncogenes, but also the role of non-clonal metabolic heterogeneity in alterating glycosylation in general.

## Conclusion

Metabolites are not only intermediate products of intracellular metabolic reactions, but also important regulators for the PTMs of cancer-related proteins like oncogenes and tumor suppressors. Emerging research demonstrates the existence of a metabolic niche within the TME, in which differing nutrient availability can mediate an indirect crosstalk between cell types and oncogenic intracellular signaling through shaping the metabolic PTMs. Targeting this novel interchange between PTMs and the environment offers new opportunities to target cancer growth in an organ-specific and more efficient manner. So far, drugs targeting modification-related enzymes such as deacetylase (HDAC) inhibitors have shown significant potential to slow down tumor growth in therapeutic settings (**Table 1**). However, at the moment, less explored PTMs as well as the interplay between the modifications and their environment lack sufficient knowledge to develop specific inhibitors. Investigating the complexity of these environmentally-driven metabolic PTMs and their dependence on tumor localization will allow us to gain a deeper mechanistic understanding and ultimately exploit these for future cancer therapies.

**Table 1:** List of oncogenes and tumor suppressors that can be regulated by post-translational modifications.

Name of modification	Oncogene/TSGs	Molecular mechanism	Cancer type	Drugs (Blocking target)
Lactylation	*HIF-1*	Up-regulates transcription of HIF-1 to modulate cancer cell proliferation and migration (28)	NSCLS	AZD3956 (MCT1)
	*YTHDF2*	Facilitates YTHDF2 expression and induces degradation of TSGs PER1 and TP53 by recognizing m6A modification sites (29)	Ocular melanoma	
	*METTL3*	Promotes Mettl3 expression and enhances its capture of m6A-modified RNA (31)	Colon cancer	
Palmitoylation	*P53*	Activates p53 signaling by promoting transcription and stabilizing its protein (51)	Breast cancer	There are no potent and specific inhibitors in clinical trials
	NRAS	Activates multiple downstream signaling pathways (48)	Leukemia	
	EGFR	Interacts with a PI3K subunit and increases reduced PI3K signaling activity (46)	Lung cancer	
	GNA13	Modulates its membrane association and signal transduction (52)	B-Cell Lymphoma	
Myristoylation	SRC	Activates Src pathways and enhances fatty-acid beta oxidation (60)	ovarian cancer	PCLX-001 (N-myristoyltransferases 1 and 2)
		Reduces its degradation and promotes plasma and endosomal membrane location (127)	B-cell lymphomas	
		Regulates its kinase activity and promotes SFK-induced oncogenic signaling (64)	prostate cancer	
	FUS1	Stabilizes Fus1 to induce apoptosis and altering cell cycle processes (65)	Lung cancer	
	EZH2	Enables it to form phase-separated droplets and liquid-like nuclear puncta; enhances interaction with STAT3 and increased STAT3 transcriptional activity (66)	Lung cancer	
Acetylation	MYC	Interacts with p30II protein, augments c-MYC-dependent transcriptional and oncogenic functions (82)	T-cell leukemia	SAHA, vorinostat (Class I and II HDACs); Romidepsin (Class I HDACs); Panobinostat (Pan-HDACs); Entinostat (Class I HDACs); Belinostat (Class I and II HDACs)
	
		Decreases its expression leading to cancer cell activation and apoptosis (128)	Acute myeloid leukemia; breast cancer	
		Enhances Myc protein stability (83)	Hepatocellular carcinoma	
		Enhances c-MYC expression to promotes proliferation and induces the apoptosis of cancer cells (129)	gastric cancer	
		Promotes the transcription of c-Myc to promote cancer cells proliferation (130)	pancreatic cancer	
		Promotes its protein level to affect cancer cell proliferation and survival (131)	Non-small cell lung cancer	
	HIF-1	Modulating the activity and protein stability of HIF-1 to regulate the balance between cell cycle arrest and apoptosis in hypoxia (132)	Osteosarcoma	
	KRAS	Enhances the stability and transcriptional activity of HIF-1α to stimulate anaerobic glycolysis (133)	Fibrosarcoma	
		Affects its activity to impact its transformative and oncogenic properties (134, 135)	Lung carcinoma, pancreatic cancer, colon cancer	
	pRb	Governs the interaction of the C-terminal E2F-1-specific domain of pRb with E2F-1 in response to DNA damage (136)	Osteosarcoma	
		Modulates its phosphorylation, protein–protein interaction and control of gene transcription (137)	Breast cancer, prostate cancer	
	p53	Enhances its stabilization to upregulate pro-apoptotic genes (138, 139)	Prostate cancer	
		Promotes its transcriptional regulation activity (140)	Breast cancer	
		Promotes the transactivation of its target genes leading to suppressed cell growth, migration and increased cell apoptosis (141)	Colorectal cancer	
		Enhances binding to PBRM1 to regulates the p53 signaling pathway (142)	Kidney cancer	
		Induces its expression and transcription-activation activities (143)	Cervical cancer	
		Increases its steady state level to induce apoptosis and autophagy cell death (144)	Endometrial cancer	
		Enhances its expression in the nucleus (145)	Lung cancer	
		Enhances its downstream apoptosis-associated genes (146)	Cutaneous T-cell lymphomas	
	PTEN	Stabilizes its expression to suppress cell growth and metastasis (147)	Laryngeal cancer	
		Induces its membrane translocation to inhibit cell migration and invasion (148)	Glioma	
		Increase its activation to suppress cell growth and invasion (149)	Hepatocellular carcinoma	
Succinylation	p53	Regulates its activation resulting in affect response to DNA damage (104)	Colorectal cancer	There are no potent and specific inhibitors in clinical trials
Glycosylation	MYC	Stabilizes its protein expression to promote cell proliferation and migration (123)	Hepatocellular carcinoma	GR-MD-02 (Galectin); GMI-1271 (Selectins); SGN-2FF (Fucosylation); GM3 (Glycolipids)
		Stabilizes its protein level to accelerate tumorigenesis (150)	Lung cancer	
	YAP	Antagonizes Hippo pathway-mediated phosphorylation of YAP (120)	liver cancer	
	HIF-1α	Delays HIF-1α degradation to regulates metabolic reprograming and survival stress signaling (151)	Breast cancer	
	EGFR	Enhances its expression and cell surface transport to regulate cell proliferation by affecting the EGFR/ERK signaling pathways (152)	Colorectal cancer	
	β-Catenin	Regulates its activity and the transcription of its downstream target genes CCND1 and MYC (153)	Pancreatic cancer	
		Increases its expression and elevates transcriptional activity (154)	Colorectal cancer	
	integrin αvβ3	Increases its sialylation to promote cell migration and invasion (111)	Breast cancer	

The regulatory molecular mechanism, cancer type affected and drug targeted therapy (if applicable) are detailed.

## Author contributions

YL, AV and PA-M: Conceptualization and Writing of the first draft. YL, AV, PA-M, S-MF: Review and editing. YL, AV, PA-M, MX, S-MF: Conceptualization and Review. All authors contributed to the article and approved the submitted version.

## Funding

AV receives funding from Fonds Wetenschappelijk Onderzoek (*FWO* 1176823N), YL receives funding from China Scholarship Council (CSC). S-MF acknowledges funding from the European Research Council under the ERC Consolidator Grant Agreement n. 771486–MetaRegulation, FWO – Research Projects (G0B4122N), KU Leuven – FTBO and Internal Funding, King Baudouin Foundation, Beug Foundation, Stichting tegen Kanker and Fonds Baillet Latour. PA-M acknowledges funding from Marie Sklodowska-Curie Actions Grant Agreement n. 839896 MetaTarGet and Beug Foundation.

## Conflict of interest

S.-MF has received funding from Bayer AG, Merck, Black Belt Therapeutics and Alesta Therapeutics; has consulted for Fund+; and serves on the advisory board of Alesta Therapeutics.

The remaining authors declare that the research was conducted in the absence of any commercial or financial relationships that could be construed as a potential conflict of interest.

## Publisher’s note

All claims expressed in this article are solely those of the authors and do not necessarily represent those of their affiliated organizations, or those of the publisher, the editors and the reviewers. Any product that may be evaluated in this article, or claim that may be made by its manufacturer, is not guaranteed or endorsed by the publisher.
